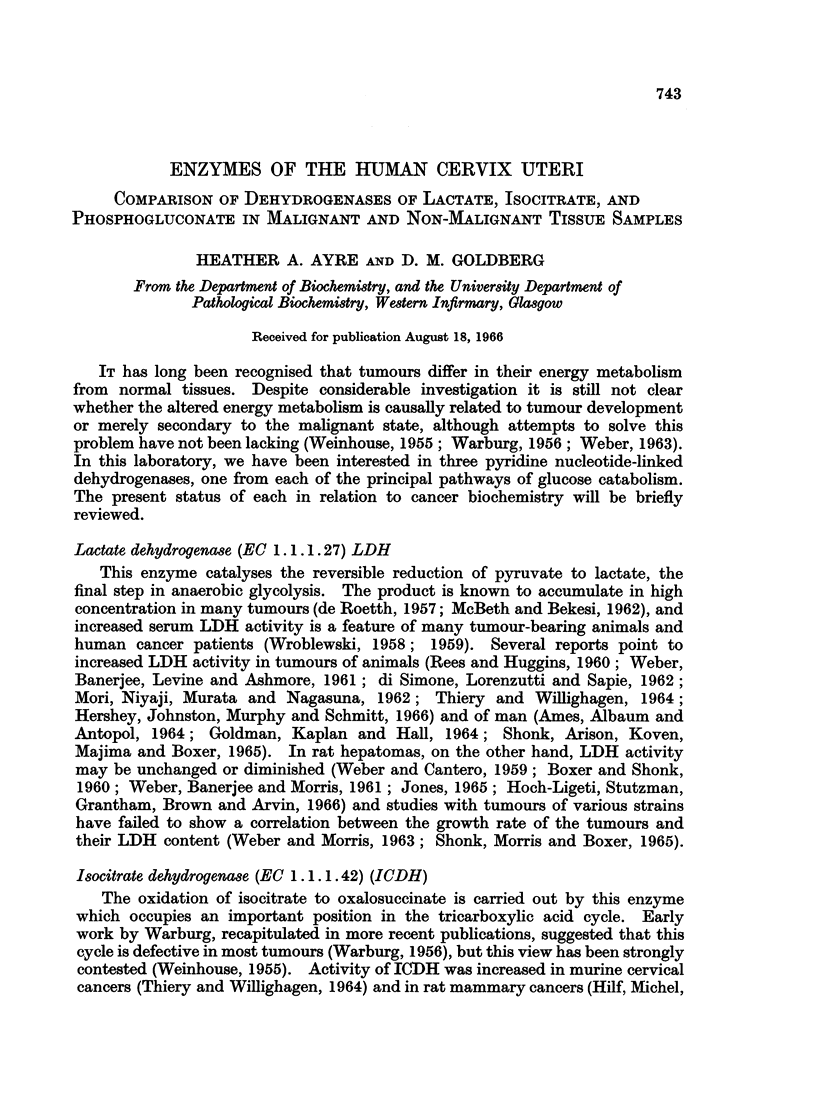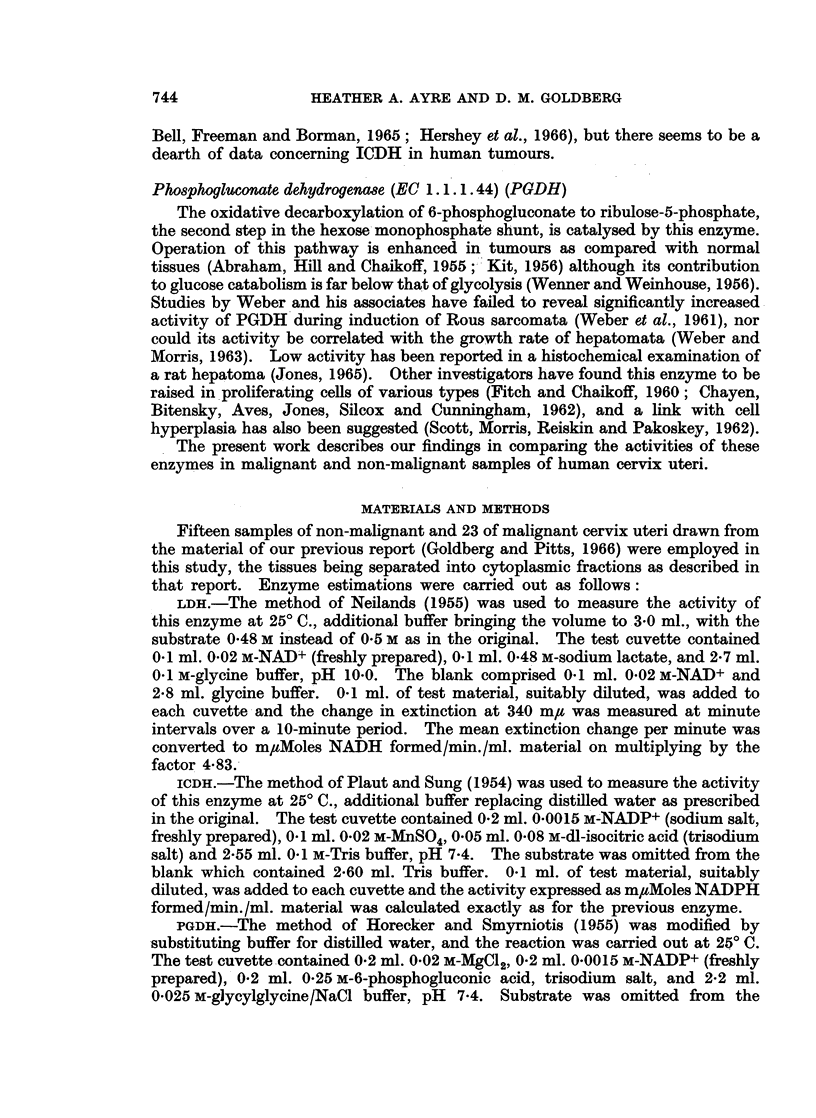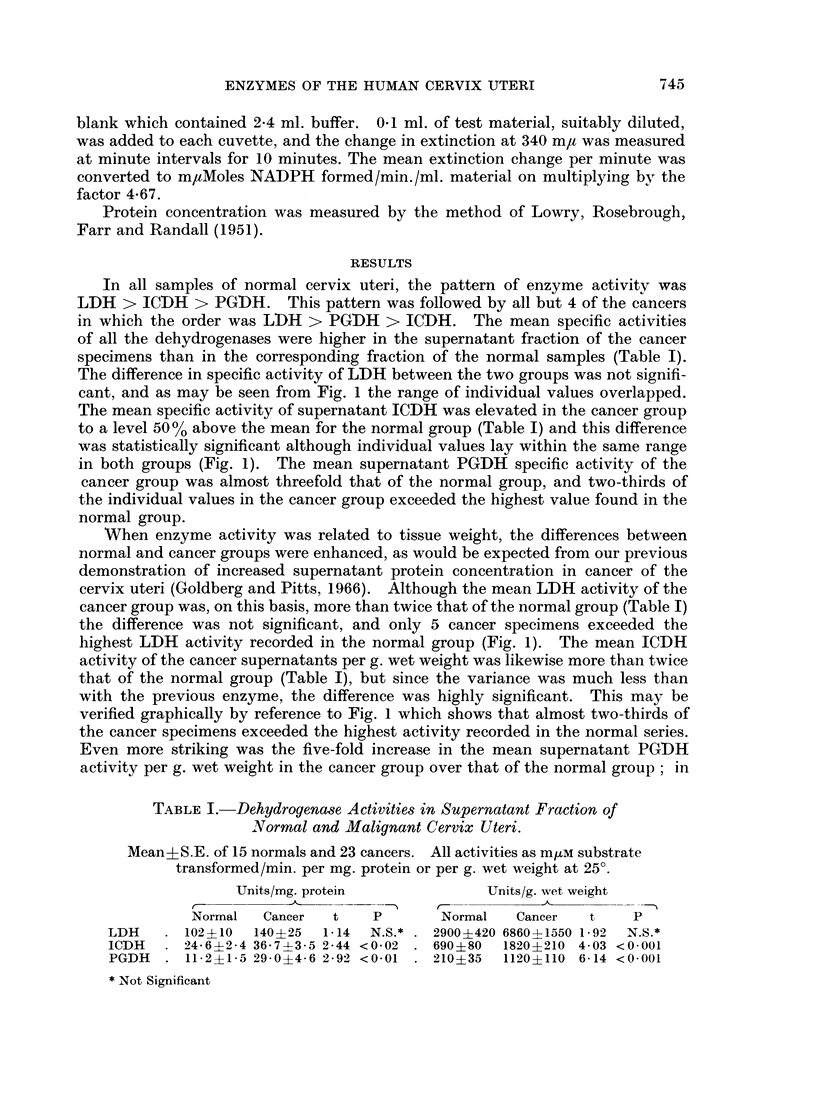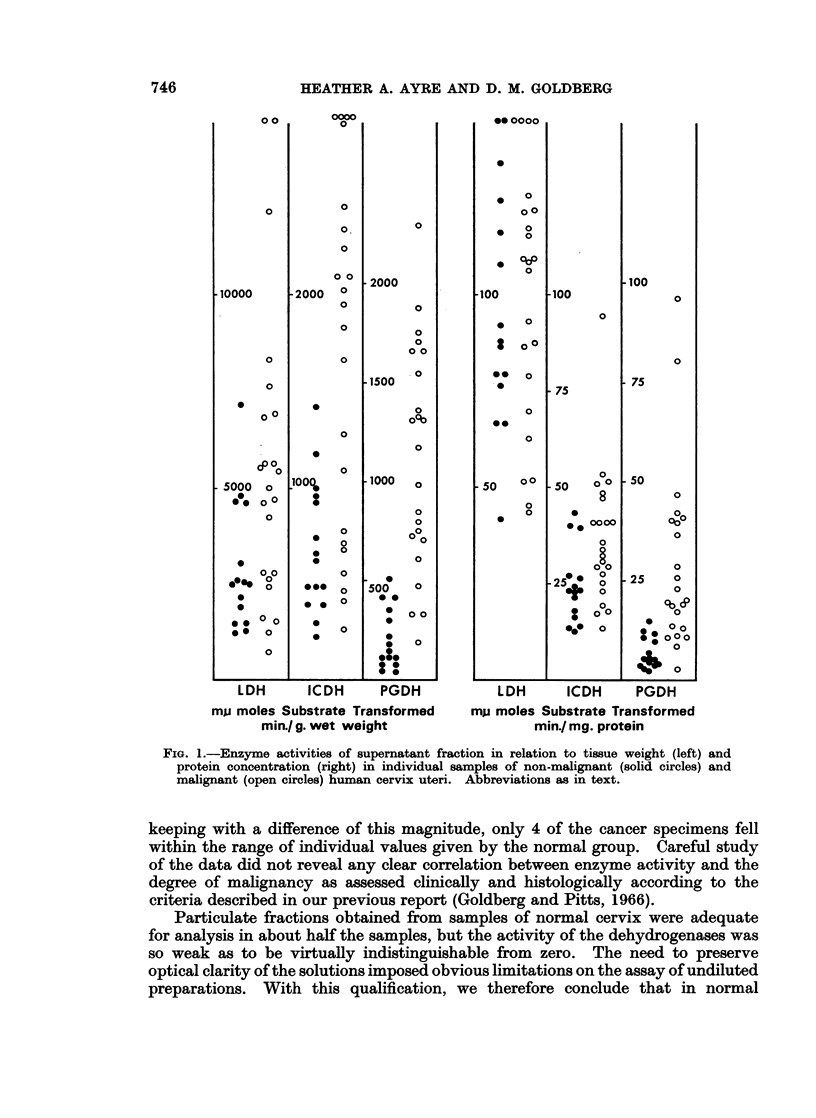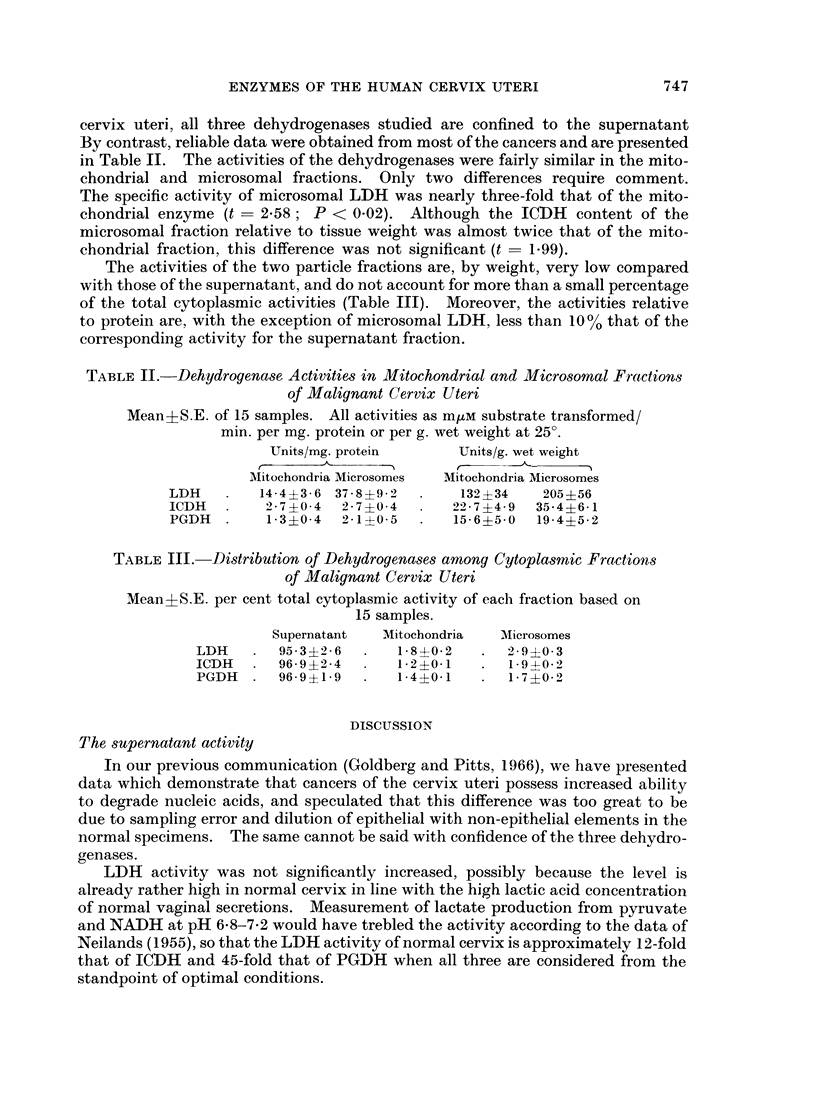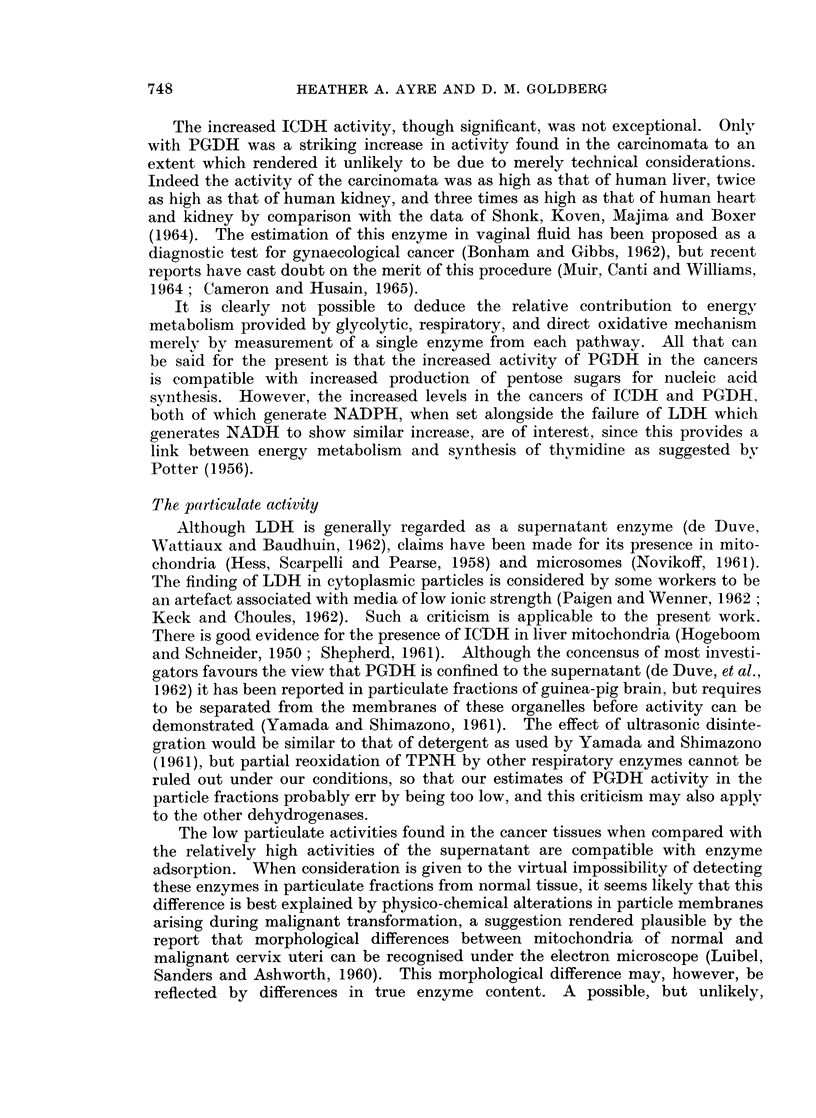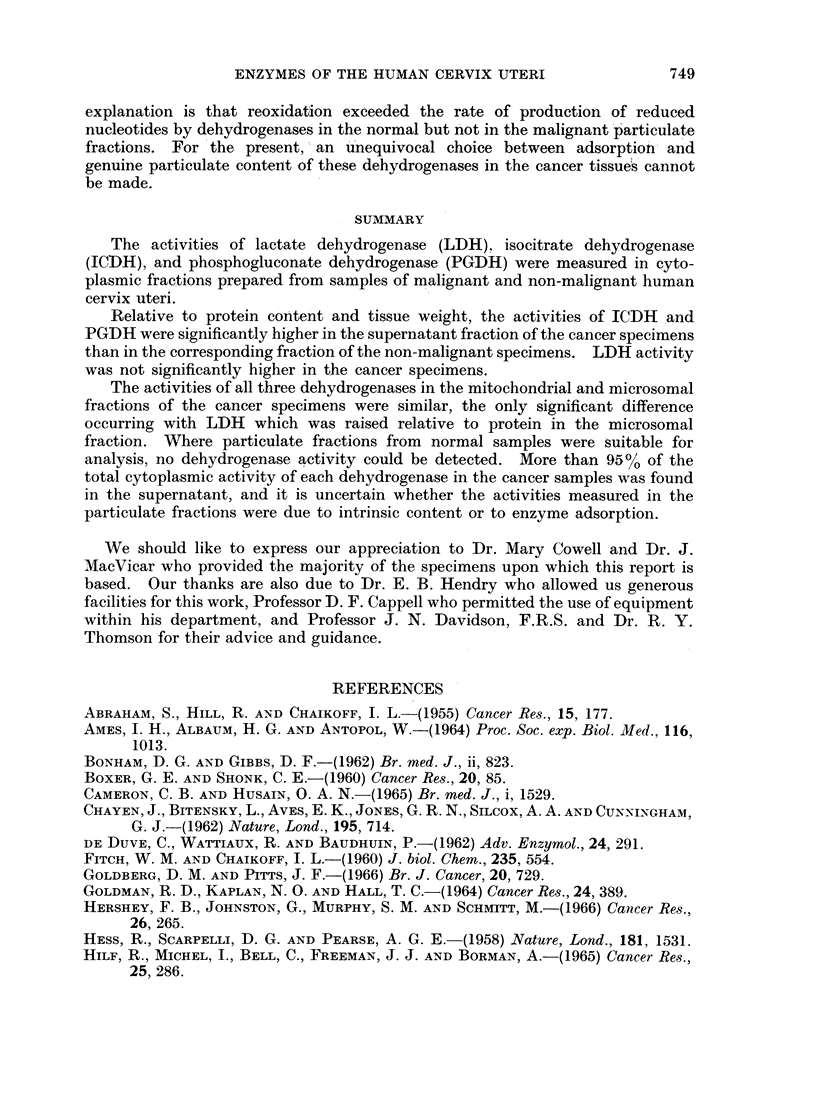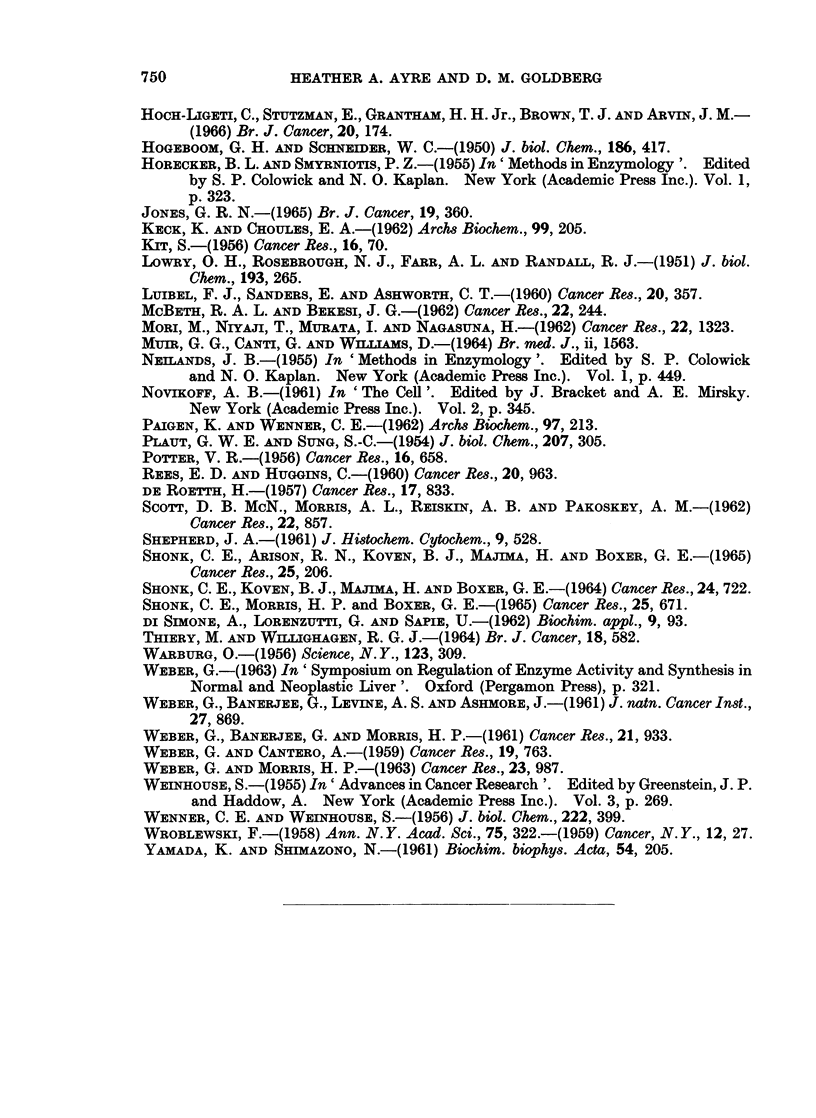# Enzymes of the human cervix uteri. Comparison of dehydrogenases of lactate, isocitrate, and phosphogluconate in malignant and non-malignant tissue samples.

**DOI:** 10.1038/bjc.1966.85

**Published:** 1966-12

**Authors:** H. A. Ayre, D. M. Goldberg


### ENZYMES OF THE HUMAN CERVIX UTERI

### Comparison of Dehydrogenases of Lactate, Isocitrate, and Phosphogluconate in Malignant and Non-Malignant Tissue Samples

HEATHER A. AYRE and D. M. GOLDBERG

*From the Department of Biochemistry, and the University Department of Pathological Biochemistry, Western Infirmary, Glasgow*

Received for publication August 18, 1966

It has long been recognised that tumours differ in their energy metabolism from normal tissues. Despite considerable investigation it is still not clear whether the altered energy metabolism is causally related to tumour development or merely secondary to the malignant state, although attempts to solve this problem have not been lacking (Weinhouse, 1955 ; Warburg, 1956 ; Weber, 1963). In this laboratory, we have been interested in three pyridine nucleotide-linked dehydrogenases, one from each of the principal pathways of glucose catabolism. The present status of each in relation to cancer biochemistry will be briefly reviewed.

*Lactate dehydrogenase* (*EC* 1.1.1.27) *LDH*

This enzyme catalyses the reversible reduction of pyruvate to lactate, the final step in anaerobic glycolysis. The product is known to accumulate in high concentration in many tumours (de Roetth, 1957; McBeth and Bekesi, 1962), and increased serum LDH activity is a feature of many tumour-bearing animals and human cancer patients (Wroblewski, 1958 ; 1959). Several reports point to increased LDH activity in tumours of animals (Rees and Huggins, 1960 ; Weber, Banerjee, Levine and Ashmore, 1961 ; di Simone, Lorenzutti and Sapie, 1962 ; Mori, Niyaji, Murata and Nagasuna, 1962 ; Thiery and Willighagen, 1964 ; Hershey, Johnston, Murphy and Schmitt, 1966) and of man (Ames, Albaum and Antopol, 1964 ; Goldman, Kaplan and Hall, 1964 ; Shonk, Arison, Koven, Majima and Boxer, 1965). In rat hepatomas, on the other hand, LDH activity may be unchanged or diminished (Weber and Cantero, 1959 ; Boxer and Shonk, 1960 ; Weber, Banerjee and Morris, 1961 ; Jones, 1965 ; Hoch-Ligeti, Stutzman, Grantham, Brown and Arvin, 1966) and studies with tumours of various strains have failed to show a correlation between the growth rate of the tumours and their LDH content (Weber and Morris, 1963 ; Shonk, Morris and Boxer, 1965).

*Isocitrate dehydrogenase* (*EC* 1.1.1.42) (*ICDH*)

The oxidation of isocitrate to oxalosuccinate is carried out by this enzyme which occupies an important position in the tricarboxylic acid cycle. Early work by Warburg, recapitulated in more recent publications, suggested that this cycle is defective in most tumours (Warburg, 1956), but this view has been strongly contested (Weinhouse, 1955). Activity of ICDH was increased in murine cervical cancers (Thiery and Willighagen, 1964) and in rat mammary cancers (Hilf, Michel,

Bell, Freeman and Borman, 1965 ; Hershey *et al.*, 1966), but there seems to be a dearth of data concerning ICDH in human tumours.

*Phosphogluconate dehydrogenase* (*EC* 1.1.1.44) (*PGDH*)

The oxidative decarboxylation of 6-phosphogluconate to ribulose-5-phosphate, the second step in the hexose monophosphate shunt, is catalysed by this enzyme. Operation of this pathway is enhanced in tumours as compared with normal tissues (Abraham, Hill and Chaikoff, 1955 ; Kit, 1956) although its contribution to glucose catabolism is far below that of glycolysis (Wenner and Weinhouse, 1956). Studies by Weber and his associates have failed to reveal significantly increased activity of PGDH during induction of Rous sarcomata (Weber *et al.*, 1961), nor could its activity be correlated with the growth rate of hepatomata (Weber and Morris, 1963). Low activity has been reported in a histochemical examination of a rat hepatoma (Jones, 1965). Other investigators have found this enzyme to be raised in proliferating cells of various types (Fitch and Chaikoff, 1960 ; Chayen, Bitensky, Aves, Jones, Silcox and Cunningham, 1962), and a link with cell hyperplasia has also been suggested (Scott, Morris, Reiskin and Pakoskey, 1962).

The present work describes our findings in comparing the activities of these enzymes in malignant and non-malignant samples of human cervix uteri.

### MATERIALS AND METHODS

Fifteen samples of non-malignant and 23 of malignant cervix uteri drawn from the material of our previous report (Goldberg and Pitts, 1966) were employed in this study, the tissues being separated into cytoplasmic fractions as described in that report. Enzyme estimations were carried out as follows:

LDH.—The method of Neilands (1955) was used to measure the activity of this enzyme at 25° C., additional buffer bringing the volume to 3·0 ml., with the substrate 0·48 M instead of 0·5 M as in the original. The test cuvette contained 0·1 ml. 0·02 M-$NAD^+$ (freshly prepared), 0·1 ml. 0·48 M-sodium lactate, and 2·7 ml. 0·1 M-glycine buffer, pH 10·0. The blank comprised 0·1 ml. 0·02 M-$NAD^+$ and 2·8 ml. glycine buffer. 0·1 ml. of test material, suitably diluted, was added to each cuvette and the change in extinction at 340 m$\mu$ was measured at minute intervals over a 10-minute period. The mean extinction change per minute was converted to m$\mu$Moles NADH formed/min./ml. material on multiplying by the factor 4·83.

ICDH.—The method of Plaut and Sung (1954) was used to measure the activity of this enzyme at 25° C., additional buffer replacing distilled water as prescribed in the original. The test cuvette contained 0·2 ml. 0·0015 M-$NADP^+$ (sodium salt, freshly prepared), 0·1 ml. 0·02 M-$MnSO_4$, 0·05 ml. 0·08 M-dl-isocitric acid (trisodium salt) and 2·55 ml. 0·1 M-Tris buffer, pH 7·4. The substrate was omitted from the blank which contained 2·60 ml. Tris buffer. 0·1 ml. of test material, suitably diluted, was added to each cuvette and the activity expressed as m$\mu$Moles NADPH formed/min./ml. material was calculated exactly as for the previous enzyme.

PGDH.—The method of Horecker and Smyrniotis (1955) was modified by substituting buffer for distilled water, and the reaction was carried out at 25° C. The test cuvette contained 0·2 ml. 0·02 M-$MgCl_2$, 0·2 ml. 0·0015 M-$NADP^+$ (freshly prepared), 0·2 ml. 0·25 M-6-phosphogluconic acid, trisodium salt, and 2·2 ml. 0·025 M-glycylglycine/NaCl buffer, pH 7·4. Substrate was omitted from the blank which contained 2·4 ml. buffer. 0·1 ml. of test material, suitably diluted, was added to each cuvette, and the change in extinction at 340 m$\mu$ was measured at minute intervals for 10 minutes. The mean extinction change per minute was converted to m$\mu$Moles NADPH formed/min./ml. material on multiplying by the factor 4·67.

Protein concentration was measured by the method of Lowry, Rosebrough, Farr and Randall (1951).

### RESULTS

In all samples of normal cervix uteri, the pattern of enzyme activity was LDH > ICDH > PGDH. This pattern was followed by all but 4 of the cancers in which the order was LDH > PGDH > ICDH. The mean specific activities of all the dehydrogenases were higher in the supernatant fraction of the cancer specimens than in the corresponding fraction of the normal samples (Table I). The difference in specific activity of LDH between the two groups was not significant, and as may be seen from Fig. 1 the range of individual values overlapped. The mean specific activity of supernatant ICDH was elevated in the cancer group to a level 50% above the mean for the normal group (Table I) and this difference was statistically significant although individual values lay within the same range in both groups (Fig. 1). The mean supernatant PGDH specific activity of the cancer group was almost threefold that of the normal group, and two-thirds of the individual values in the cancer group exceeded the highest value found in the normal group.

When enzyme activity was related to tissue weight, the differences between normal and cancer groups were enhanced, as would be expected from our previous demonstration of increased supernatant protein concentration in cancer of the cervix uteri (Goldberg and Pitts, 1966). Although the mean LDH activity of the cancer group was, on this basis, more than twice that of the normal group (Table I) the difference was not significant, and only 5 cancer specimens exceeded the highest LDH activity recorded in the normal group (Fig. 1). The mean ICDH activity of the cancer supernatants per g. wet weight was likewise more than twice that of the normal group (Table I), but since the variance was much less than with the previous enzyme, the difference was highly significant. This may be verified graphically by reference to Fig. 1 which shows that almost two-thirds of the cancer specimens exceeded the highest activity recorded in the normal series. Even more striking was the five-fold increase in the mean supernatant PGDH activity per g. wet weight in the cancer group over that of the normal group ; in

TABLE I.—*Dehydrogenase Activities in Supernatant Fraction of Normal and Malignant Cervix Uteri.*

Mean$\pm$S.E. of 15 normals and 23 cancers. All activities as m$\mu$M substrate transformed/min. per mg. protein or per g. wet weight at 25°.

| | Units/mg. protein | | | | Units/g. wet weight | | | |
|---|---|---|---|---|---|---|---|---|
| | Normal | Cancer | t | P | Normal | Cancer | t | P |
| LDH | 102$\pm$10 | 140$\pm$25 | 1·14 | N.S.* | 2900$\pm$420 | 6860$\pm$1550 | 1·92 | N.S.* |
| ICDH | 24·6$\pm$2·4 | 36·7$\pm$3·5 | 2·44 | <0·02 | 690$\pm$80 | 1820$\pm$210 | 4·03 | <0·001 |
| PGDH | 11·2$\pm$1·5 | 29·0$\pm$4·6 | 2·92 | <0·01 | 210$\pm$35 | 1120$\pm$110 | 6·14 | <0·001 |

* Not Significant

FIG. 1.—Enzyme activities of supernatant fraction in relation to tissue weight (left) and protein concentration (right) in individual samples of non-malignant (solid circles) and malignant (open circles) human cervix uteri. Abbreviations as in text.

keeping with a difference of this magnitude, only 4 of the cancer specimens fell within the range of individual values given by the normal group. Careful study of the data did not reveal any clear correlation between enzyme activity and the degree of malignancy as assessed clinically and histologically according to the criteria described in our previous report (Goldberg and Pitts, 1966).

Particulate fractions obtained from samples of normal cervix were adequate for analysis in about half the samples, but the activity of the dehydrogenases was so weak as to be virtually indistinguishable from zero. The need to preserve optical clarity of the solutions imposed obvious limitations on the assay of undiluted preparations. With this qualification, we therefore conclude that in normal cervix uteri, all three dehydrogenases studied are confined to the supernatant By contrast, reliable data were obtained from most of the cancers and are presented in Table II. The activities of the dehydrogenases were fairly similar in the mitochondrial and microsomal fractions. Only two differences require comment. The specific activity of microsomal LDH was nearly three-fold that of the mitochondrial enzyme ($t = 2{\cdot}58$ ; $P < 0{\cdot}02$). Although the ICDH content of the microsomal fraction relative to tissue weight was almost twice that of the mitochondrial fraction, this difference was not significant ($t = 1{\cdot}99$).

The activities of the two particle fractions are, by weight, very low compared with those of the supernatant, and do not account for more than a small percentage of the total cytoplasmic activities (Table III). Moreover, the activities relative to protein are, with the exception of microsomal LDH, less than 10% that of the corresponding activity for the supernatant fraction.

TABLE II.—*Dehydrogenase Activities in Mitochondrial and Microsomal Fractions of Malignant Cervix Uteri*

Mean±S.E. of 15 samples. All activities as mμM substrate transformed/min. per mg. protein or per g. wet weight at 25°.

| | Units/mg. protein | | Units/g. wet weight | |
|---|---|---|---|---|
| | Mitochondria | Microsomes | Mitochondria | Microsomes |
| LDH | 14·4±3·6 | 37·8±9·2 | 132±34 | 205±56 |
| ICDH | 2·7±0·4 | 2·7±0·4 | 22·7±4·9 | 35·4±6·1 |
| PGDH | 1·3±0·4 | 2·1±0·5 | 15·6±5·0 | 19·4±5·2 |

TABLE III.—*Distribution of Dehydrogenases among Cytoplasmic Fractions of Malignant Cervix Uteri*

Mean±S.E. per cent total cytoplasmic activity of each fraction based on 15 samples.

| | Supernatant | Mitochondria | Microsomes |
|---|---|---|---|
| LDH | 95·3±2·6 | 1·8±0·2 | 2·9±0·3 |
| ICDH | 96·9±2·4 | 1·2±0·1 | 1·9±0·2 |
| PGDH | 96·9±1·9 | 1·4±0·1 | 1·7±0·2 |

### DISCUSSION

### *The supernatant activity*

In our previous communication (Goldberg and Pitts, 1966), we have presented data which demonstrate that cancers of the cervix uteri possess increased ability to degrade nucleic acids, and speculated that this difference was too great to be due to sampling error and dilution of epithelial with non-epithelial elements in the normal specimens. The same cannot be said with confidence of the three dehydrogenases.

LDH activity was not significantly increased, possibly because the level is already rather high in normal cervix in line with the high lactic acid concentration of normal vaginal secretions. Measurement of lactate production from pyruvate and NADH at pH 6·8–7·2 would have trebled the activity according to the data of Neilands (1955), so that the LDH activity of normal cervix is approximately 12-fold that of ICDH and 45-fold that of PGDH when all three are considered from the standpoint of optimal conditions.

The increased ICDH activity, though significant, was not exceptional. Only with PGDH was a striking increase in activity found in the carcinomata to an extent which rendered it unlikely to be due to merely technical considerations. Indeed the activity of the carcinomata was as high as that of human liver, twice as high as that of human kidney, and three times as high as that of human heart and kidney by comparison with the data of Shonk, Koven, Majima and Boxer (1964). The estimation of this enzyme in vaginal fluid has been proposed as a diagnostic test for gynaecological cancer (Bonham and Gibbs, 1962), but recent reports have cast doubt on the merit of this procedure (Muir, Canti and Williams, 1964 ; Cameron and Husain, 1965).

It is clearly not possible to deduce the relative contribution to energy metabolism provided by glycolytic, respiratory, and direct oxidative mechanism merely by measurement of a single enzyme from each pathway. All that can be said for the present is that the increased activity of PGDH in the cancers is compatible with increased production of pentose sugars for nucleic acid synthesis. However, the increased levels in the cancers of ICDH and PGDH, both of which generate NADPH, when set alongside the failure of LDH which generates NADH to show similar increase, are of interest, since this provides a link between energy metabolism and synthesis of thymidine as suggested by Potter (1956).

*The particulate activity*

Although LDH is generally regarded as a supernatant enzyme (de Duve, Wattiaux and Baudhuin, 1962), claims have been made for its presence in mitochondria (Hess, Scarpelli and Pearse, 1958) and microsomes (Novikoff, 1961). The finding of LDH in cytoplasmic particles is considered by some workers to be an artefact associated with media of low ionic strength (Paigen and Wenner, 1962 ; Keck and Choules, 1962). Such a criticism is applicable to the present work. There is good evidence for the presence of ICDH in liver mitochondria (Hogeboom and Schneider, 1950 ; Shepherd, 1961). Although the concensus of most investigators favours the view that PGDH is confined to the supernatant (de Duve, *et al.*, 1962) it has been reported in particulate fractions of guinea-pig brain, but requires to be separated from the membranes of these organelles before activity can be demonstrated (Yamada and Shimazono, 1961). The effect of ultrasonic disintegration would be similar to that of detergent as used by Yamada and Shimazono (1961), but partial reoxidation of TPNH by other respiratory enzymes cannot be ruled out under our conditions, so that our estimates of PGDH activity in the particle fractions probably err by being too low, and this criticism may also apply to the other dehydrogenases.

The low particulate activities found in the cancer tissues when compared with the relatively high activities of the supernatant are compatible with enzyme adsorption. When consideration is given to the virtual impossibility of detecting these enzymes in particulate fractions from normal tissue, it seems likely that this difference is best explained by physico-chemical alterations in particle membranes arising during malignant transformation, a suggestion rendered plausible by the report that morphological differences between mitochondria of normal and malignant cervix uteri can be recognised under the electron microscope (Luibel, Sanders and Ashworth, 1960). This morphological difference may, however, be reflected by differences in true enzyme content. A possible, but unlikely, explanation is that reoxidation exceeded the rate of production of reduced nucleotides by dehydrogenases in the normal but not in the malignant particulate fractions. For the present, an unequivocal choice between adsorption and genuine particulate content of these dehydrogenases in the cancer tissues cannot be made.

### SUMMARY

The activities of lactate dehydrogenase (LDH), isocitrate dehydrogenase (ICDH), and phosphogluconate dehydrogenase (PGDH) were measured in cytoplasmic fractions prepared from samples of malignant and non-malignant human cervix uteri.

Relative to protein content and tissue weight, the activities of ICDH and PGDH were significantly higher in the supernatant fraction of the cancer specimens than in the corresponding fraction of the non-malignant specimens. LDH activity was not significantly higher in the cancer specimens.

The activities of all three dehydrogenases in the mitochondrial and microsomal fractions of the cancer specimens were similar, the only significant difference occurring with LDH which was raised relative to protein in the microsomal fraction. Where particulate fractions from normal samples were suitable for analysis, no dehydrogenase activity could be detected. More than 95% of the total cytoplasmic activity of each dehydrogenase in the cancer samples was found in the supernatant, and it is uncertain whether the activities measured in the particulate fractions were due to intrinsic content or to enzyme adsorption.

We should like to express our appreciation to Dr. Mary Cowell and Dr. J. MacVicar who provided the majority of the specimens upon which this report is based. Our thanks are also due to Dr. E. B. Hendry who allowed us generous facilities for this work, Professor D. F. Cappell who permitted the use of equipment within his department, and Professor J. N. Davidson, F.R.S. and Dr. R. Y. Thomson for their advice and guidance.

### REFERENCES

Abraham, S., Hill, R. and Chaikoff, I. L.—(1955) *Cancer Res.*, **15**, 177.

Ames, I. H., Albaum, H. G. and Antopol, W.—(1964) *Proc. Soc. exp. Biol. Med.*, **116**, 1013.

Bonham, D. G. and Gibbs, D. F.—(1962) *Br. med. J.*, ii, 823.

Boxer, G. E. and Shonk, C. E.—(1960) *Cancer Res.*, **20**, 85.

Cameron, C. B. and Husain, O. A. N.—(1965) *Br. med. J.*, i, 1529.

Chayen, J., Bitensky, L., Aves, E. K., Jones, G. R. N., Silcox, A. A. and Cunningham, G. J.—(1962) *Nature, Lond.*, **195**, 714.

de Duve, C., Wattiaux, R. and Baudhuin, P.—(1962) *Adv. Enzymol.*, **24**, 291.

Fitch, W. M. and Chaikoff, I. L.—(1960) *J. biol. Chem.*, **235**, 554.

Goldberg, D. M. and Pitts, J. F.—(1966) *Br. J. Cancer*, **20**, 729.

Goldman, R. D., Kaplan, N. O. and Hall, T. C.—(1964) *Cancer Res.*, **24**, 389.

Hershey, F. B., Johnston, G., Murphy, S. M. and Schmitt, M.—(1966) *Cancer Res.*, **26**, 265.

Hess, R., Scarpelli, D. G. and Pearse, A. G. E.—(1958) *Nature, Lond.*, **181**, 1531.

Hilf, R., Michel, I., Bell, C., Freeman, J. J. and Borman, A.—(1965) *Cancer Res.*, **25**, 286.

HOCH-LIGETI, C., STUTZMAN, E., GRANTHAM, H. H. Jr., BROWN, T. J. AND ARVIN, J. M.—(1966) *Br. J. Cancer*, **20**, 174.
HOGEBOOM, G. H. AND SCHNEIDER, W. C.—(1950) *J. biol. Chem.*, **186**, 417.
HORECKER, B. L. AND SMYRNIOTIS, P. Z.—(1955) *In* ' Methods in Enzymology '. Edited by S. P. Colowick and N. O. Kaplan. New York (Academic Press Inc.). Vol. 1, p. 323.
JONES, G. R. N.—(1965) *Br. J. Cancer*, **19**, 360.
KECK, K. AND CHOULES, E. A.—(1962) *Archs Biochem.*, **99**, 205.
KIT, S.—(1956) *Cancer Res.*, **16**, 70.
LOWRY, O. H., ROSEBROUGH, N. J., FARR, A. L. AND RANDALL, R. J.—(1951) *J. biol. Chem.*, **193**, 265.
LUIBEL, F. J., SANDERS, E. AND ASHWORTH, C. T.—(1960) *Cancer Res.*, **20**, 357.
McBETH, R. A. L. AND BEKESI, J. G.—(1962) *Cancer Res.*, **22**, 244.
MORI, M., NIYAJI, T., MURATA, I. AND NAGASUNA, H.—(1962) *Cancer Res.*, **22**, 1323.
MUIR, G. G., CANTI, G. AND WILLIAMS, D.—(1964) *Br. med. J.*, ii, 1563.
NEILANDS, J. B.—(1955) *In* ' Methods in Enzymology '. Edited by S. P. Colowick and N. O. Kaplan. New York (Academic Press Inc.). Vol. 1, p. 449.
NOVIKOFF, A. B.—(1961) *In* ' The Cell '. Edited by J. Bracket and A. E. Mirsky. New York (Academic Press Inc.). Vol. 2, p. 345.
PAIGEN, K. AND WENNER, C. E.—(1962) *Archs Biochem.*, **97**, 213.
PLAUT, G. W. E. AND SUNG, S.-C.—(1954) *J. biol. Chem.*, **207**, 305.
POTTER, V. R.—(1956) *Cancer Res.*, **16**, 658.
REES, E. D. AND HUGGINS, C.—(1960) *Cancer Res.*, **20**, 963.
DE ROETTH, H.—(1957) *Cancer Res.*, **17**, 833.
SCOTT, D. B. McN., MORRIS, A. L., REISKIN, A. B. AND PAKOSKEY, A. M.—(1962) *Cancer Res.*, **22**, 857.
SHEPHERD, J. A.—(1961) *J. Histochem. Cytochem.*, **9**, 528.
SHONK, C. E., ARISON, R. N., KOVEN, B. J., MAJIMA, H. AND BOXER, G. E.—(1965) *Cancer Res.*, **25**, 206.
SHONK, C. E., KOVEN, B. J., MAJIMA, H. AND BOXER, G. E.—(1964) *Cancer Res.*, **24**, 722.
SHONK, C. E., MORRIS, H. P. and BOXER, G. E.—(1965) *Cancer Res.*, **25**, 671.
DI SIMONE, A., LORENZUTTI, G. AND SAPIE, U.—(1962) *Biochim. appl.*, **9**, 93.
THIERY, M. AND WILLIGHAGEN, R. G. J.—(1964) *Br. J. Cancer*, **18**, 582.
WARBURG, O.—(1956) *Science, N.Y.*, **123**, 309.
WEBER, G.—(1963) *In* ' Symposium on Regulation of Enzyme Activity and Synthesis in Normal and Neoplastic Liver '. Oxford (Pergamon Press), p. 321.
WEBER, G., BANERJEE, G., LEVINE, A. S. AND ASHMORE, J.—(1961) *J. natn. Cancer Inst.*, **27**, 869.
WEBER, G., BANERJEE, G. AND MORRIS, H. P.—(1961) *Cancer Res.*, **21**, 933.
WEBER, G. AND CANTERO, A.—(1959) *Cancer Res.*, **19**, 763.
WEBER, G. AND MORRIS, H. P.—(1963) *Cancer Res.*, **23**, 987.
WEINHOUSE, S.—(1955) *In* ' Advances in Cancer Research '. Edited by Greenstein, J. P. and Haddow, A. New York (Academic Press Inc.). Vol. 3, p. 269.
WENNER, C. E. AND WEINHOUSE, S.—(1956) *J. biol. Chem.*, **222**, 399.
WROBLEWSKI, F.—(1958) *Ann. N.Y. Acad. Sci.*, **75**, 322.—(1959) *Cancer, N.Y.*, **12**, 27.
YAMADA, K. AND SHIMAZONO, N.—(1961) *Biochim. biophys. Acta*, **54**, 205.